# On-Chip Temperature Compensation for Small-Signal Gain Variation Reduction

**DOI:** 10.3390/mi13071101

**Published:** 2022-07-13

**Authors:** Shaohua Zhou, Shizhe Wei, Jian Wang

**Affiliations:** 1 School of Microelectronics, Tianjin University, Tianjin 300072, China; zhoushaohua@tju.edu.cn (S.Z.); shizhe_wei@vanchip.com (S.W.); 2 Qingdao Institute for Ocean Technology, Tianjin University, Qingdao 266200, China

**Keywords:** millimeter-wave, CMOS, power amplifier, specification degradation, temperature compensation

## Abstract

Power amplifier (PA) specifications are closely related to changes in temperature; thus, the small-signal gain (S21) of PA decreases with the temperature increase. To compensate for the degradation caused by the decrease in S21, we present a compensation circuit that consists of two diodes and four resistors. At the same time, a differential stacked millimeter-wave wideband PA was designed and implemented based on this compensation circuit and 55 nm CMOS process. The post-layout simulation results showed that the fluctuation of S21 reduced from 2.4 dB to 0.1 dB in the frequency range of 25−40 GHz over the temperature range of −40 °C to 125 °C. Furthermore, the proposed on-chip temperature compensation circuit also applies to multi-stage cascaded microwave/mm-wave power amplifiers.

## 1. Introduction

In today’s highly developed communication industry, the spectrum resources for the low-frequency band are becoming increasingly scarce, which seriously restricts the development of the wireless communication industry. The millimeter-wave band has received much attention because of the abundant and unexplored spectrum resources. A PA is a key module of a millimeter-wave wireless communication system [1,2,3], and its performance directly determines the quality of a wireless communication system. Millimeter-wave communication systems, for example, wireless communication [4,5,6,7], radars [8,9,10], satellites [11,12], and navigation [13], usually operate in outdoor or extremely harsh places with significant temperature variations, and the specifications of PAs are closely related to the temperature variation. Temperature variation leads to the specification degradation of PAs [14,15,16], which makes the millimeter-wave wireless communication system unable to meet the normal operation requirements or even fail [17,18,19,20]. Therefore, reducing the effect of temperature change on the PA’s specification degradation under the dynamic, temperature-changing operating environment has become an urgent problem.

To address the above issues, a lot of fruitful work has been carried out by domestic and international researchers. For example, in 2017, Javed S. Gaggatur et al. from the Indian Institute of Science and Technology proposed a technique for the performance compensation of integrated CMOS power amplifiers based on non-invasive temperature sensing, which is expected to compensate for degradation due to self-heating [21]. A new method for the temperature compensation of on-chip differential logarithmic amplifiers was proposed by Y. Wenger of the Technical University of Braunschweig, Germany, and A. Ghazinour of NXP Semiconductors in 2018 [22]. The results showed that better compensation is achieved in the temperature range of −40 °C to 125 °C. In 2018, Zhiming Chen et al. of the Beijing Institute of Technology proposed a new temperature compensation method for K-band CMOS amplifiers [23], which achieves better compensation for PA gain in the temperature range of −45 °C to +125 °C. In 2020, Fariborz Lohrabi Pour et al. from Virginia Tech proposed a temperature-compensated power amplifier [24] that can operate reliably over a temperature range of −40 °C to +225 °C.

In summary, various temperature compensation techniques and methods have been successfully applied in the research and design of various single-stage and multi-stage power amplifiers. Still, little research has been reported on the temperature compensation of power amplifiers with stacked structures. However, the stacked structure is widely used to implement various high-output power PAs, because it increases the amplifier’s output power [25].

Therefore, this paper presents a temperature compensation circuit for a stacked power amplifier consisting of only two diodes and four resistors. The proposed compensation circuit keeps the gain constant with the temperature by controlling the gate voltage. In addition, the compensation circuit uses the principle that the diode’s threshold voltage decreases with the temperature increase. This study first investigated the temperature compensation circuit’s operating principle and design method. Then, based on the proposed on-chip temperature compensation circuit and 55 nm CMOS technology, a differential stacked millimeter-wave wideband PA was implemented. As a result, the power amplifier’s small-signal gain fluctuated from 2.4 dB to 0.1 dB in the temperature range of −40 °C to +125 °C, and the frequency range was 25−40 GHz.

## 2. Temperature Characteristics of Single-Stage Amplifier Gain

**Figure 1:**
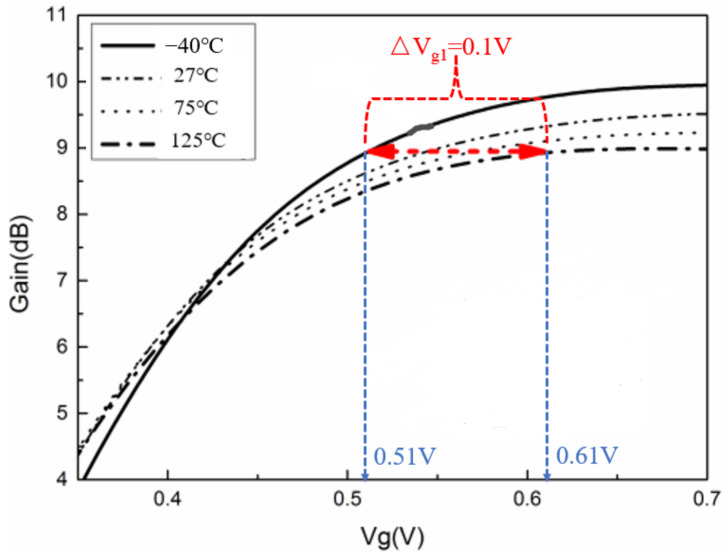
Temperature characteristics of gain of single-stage PA.

The gain of a single-stage PA decreases with increasing temperature [26], while the gain of a class AB PA increases with increasing gate voltage [27]. Therefore, the gain of the amplifier can be compensated by controlling the transistor gate voltage. The temperature characteristics of the gain of a single-stage PA are shown in Figure 1. When the gate voltage changes between 0.51 V and 0.61 V, the gain of the single-stage power amplifier remains unchanged in the range of −40 °C to +125 °C.

The transconductance in the saturated regions [28,29,30]:

(1) $$g_{ms}=\frac{\partial I_{D}(sat)}{\partial V_{GS}}=\mu_{n}(T_{0}){\left(\frac{T}{T_{0}}\right)}^{-3/2}\frac{WC_{ox}}{L}(V_{GS}-V_{T}),$$

where *W* is the gate width, *μ_n_* is the carrier mobility, *C_ox_* is the gate oxide capacitance per unit area, *L* is the gate length, *V_GS_* is the gate voltage, *V_T_* is the threshold voltage, *V_DS_* is the drain voltage, and *T*_0_ = 300 K.

Typically, the saturation voltage (*V_GS_*-*V_T_*) is chosen to be relatively large in order to obtain a large transconductance, so that the effect of the threshold voltage can be neglected. Moreover, its influence tends to dominate due to the exponential nature of carrier mobility [30]. Therefore, the transconductance usually decreases with increasing temperature, and the transconductance is usually considered as the gain of the transistor [29]. Consequently, the small-signal gain of the PA decreases with increasing temperature.

## 3. Principle and Design of Temperature Compensation Circuit

**Figure 2:**
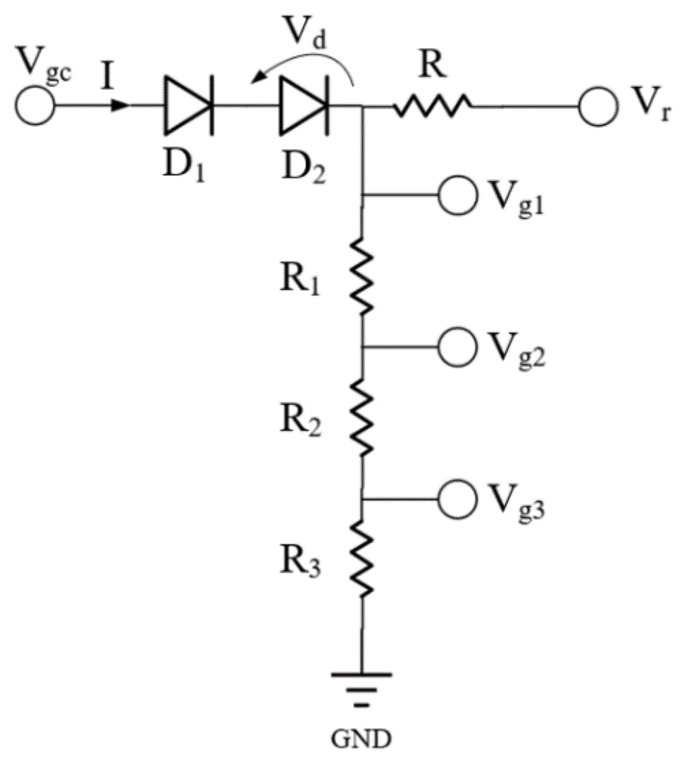
Schematic diagram of the compensation circuit.

As described in Section 1, increasing the gate voltage can compensate for the change in amplifier gain. This temperature compensation circuit consists of two diodes and four resistors, as shown in Figure 2. The values of gate voltages *V_g_*_1_, *V_g_*_2_, and *V_g_*_3_ are determined by the *V_r_* and *V_gc_*. In Figure 2, *D*_1_ and *D*_2_ are diodes, *R*, *R*_1_, *R*_2_, and *R*_3_ are resistors, and *V_d_* and *I* are the current and voltage through the diodes, respectively. The compensation circuit shown in Figure 2 utilizes the mechanism whereby the threshold voltage of the diode decreases with increasing temperature.

To facilitate the presentation of the operating principle of the temperature compensation circuit, it is assumed that *V_gc_* = 0. The following equation can be derived from Figure 2 [31].

(2) $$-V_{r}=RI+2V_{d},$$

(3) $$I=I_{s}\left\{\exp(\frac{qV_{d}}{nkT})-1\right\},$$

where *T* is the temperature, and *n* is the ideal coefficient of the diode. *I_s_* can be expressed as

(4) $$I_{s}=SA^{*}T^{2}\exp(-\frac{q\phi_{B}}{kT}),$$

where A* is the temperature-independent Richardson–Dushman constant, *φ_B_* is the material-dependent Schottky barrier voltage, and *S* is the junction size of the diode. From (2), (3), and (4), it follows that *V_g_*_1_ is a function of *T*:

(5) $$V_{g1}=2\left\{\frac{kT}{q}\ln(SRA^{*})+\frac{2kT}{q}\ln(T)-\phi_{B}\right\}n,$$

where *V_g_*_1_ = −2*V_d_* (as shown in Figure 2).

Assume that *RI_s_* ≪ −(*V_r_* + 2*V_d_*) ≪ (1/*RI_s_*). Then, from (4), the following equation can be derived.

(6) $$\frac{\partial V_{g1}}{\partial T}=2\frac{nk}{q}\left\{\ln(SRA^{*})+2\ln(T)+2\right\},$$

If *T* is greater than −270 °C, we have *∂V_g_*_1_/*∂T* > 0. *V_g_*_1_ increases with increasing temperature for *T* greater than −270 °C. The following equation can calculate the variation of *V_g_*_1_ (Δ*V_g_*_1_) between two temperatures, *T_H_* and *T_L_*.

(7) $$\begin{array}{l} \Delta V_{g1}=\int_{T_{L}}^{T_{H}}\frac{\partial V_{g1}}{\partial T}dT=\int_{T_{L}}^{T_{H}}2\frac{nk}{q}\left\{\ln(SRA^{*})+2\ln(T)+2\right\}dT\\ =\frac{2nk}{q}(T_{H}-T_{L})\ln(SRA^{*})+\frac{4nk}{q}(T_{H}ln(T_{H})-T_{L}ln(T_{L})) \end{array}$$

Therefore, the expressions for *V_g_*_2_ and *V_g_*_3_ can be obtained according to Figure 2 and Equation (5).

(8) $$V_{g2}=V_{g1}\frac{R_{2}+R_{3}}{R_{1}+R_{2}+R_{3}},$$

(9) $$V_{g3}=V_{g1}\frac{R_{3}}{R_{1}+R_{2}+R_{3}},$$

According to (8) and (9), *V_g_*_2_ and *V_g_*_3_ are proportional to *V_g_*_1_, increasing with temperature. Therefore, *V_g_*_2_ and *V_g_*_3_ also increase with temperature in order to realize gain compensation. The following explains the compensation circuit’s principle to compensate for the PA’s small-signal gain.

When the temperature increases, gate voltage *V_g_*_1_, gate voltage *V_g_*_2_, and gate voltage *V_g_*_3_ in the temperature compensation circuit all increase as the temperature increases. The increase in gate voltage *V_g_*_1_, gate voltage *V_g_*_2_, and gate voltage *V_g_*_3_ is equivalent to the rise in the magnitude of *V_GS_* of Equation (1). When *V_g_*_1_, *V_g_*_2_, and *V_g_*_3_ increase with temperature, the transconductance of each layer of the stacked PA can be expressed as

(10) $$g_{1}=\frac{\partial I_{D}(sat)}{\partial V_{GS}}=\mu_{n}(T_{0}){\left(\frac{T}{T_{0}}\right)}^{-3/2}\frac{WC_{ox}}{L}(V_{g1}+\Delta V_{g1}-V_{T}),$$

(11) $$g_{2}=\frac{\partial I_{D}(sat)}{\partial V_{GS}}=\mu_{n}(T_{0}){\left(\frac{T}{T_{0}}\right)}^{-3/2}\frac{WC_{ox}}{L}(V_{g2}+\Delta V_{g2}-V_{T}),$$

(12) $$g_{3}=\frac{\partial I_{D}(sat)}{\partial V_{GS}}=\mu_{n}(T_{0}){\left(\frac{T}{T_{0}}\right)}^{-3/2}\frac{WC_{ox}}{L}(V_{g3}+\Delta V_{g3}-V_{T}),$$

According to Equations (10)–(12), the transconductance (i.e., gain) of each layer increases with Δ*V_g_*_1_, Δ*V_g_*_2_, and Δ*V_g_*_3_, i.e., with the increase in temperature. Thus, the compensation for the degradation of the gain of each layer with the increase in temperature is achieved.

It should be noted that the on-chip temperature compensation circuit described in this section does not have any additional requirements or constraints on the design of the PA. In addition, the compensation circuit was designed for a single-stage PA. Specifically, for a multi-stage PA, the parameters of each transistor stage are different. Then, it is necessary to determine the value of Δ*V_g_*, similar to the one shown in Figure 1, based on the parameters of each transistor stage. From Equation (7), it can be seen that the parameters affecting ΔV_g1_ are mainly the junction size (S) and resistance (R) of the diode. Therefore, for a multi-stage PA, the design of the corresponding temperature compensation circuit can be completed by simply adjusting the values of S and R so that the Δ*V_g_*_1_ (as shown in Equation (7)) of each stage meets the requirements similar to the Δ*V_g_*_1_ in Figure 1.

## 4. Differential Stacked Millimeter-Wave Broadband PA

**Figure 3:**
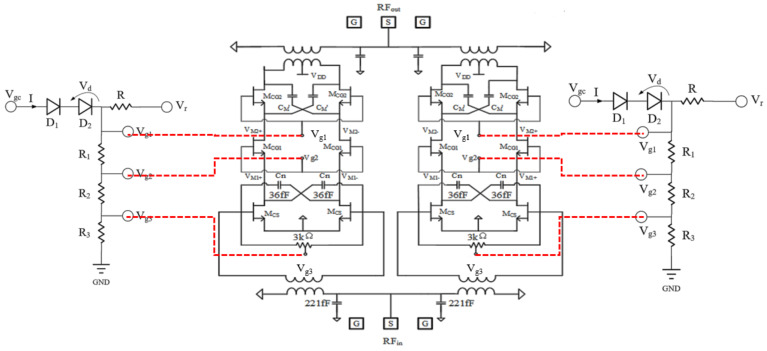
Schematic diagram of differential stacked PA, based on temperature compensation circuit.

To verify the effectiveness of the proposed on-chip temperature compensation circuit, a differential stacked millimeter-wave wideband PA was implemented in this study. Figure 3 shows the schematic of a differential stacked millimeter-wave wideband PA, which is used to compensate for the degradation of small-signal gain. As shown in Figure 1, the required Δ*V_g_*_1_ is 0.1 V. Two diodes are connected in series to achieve Δ*V_g_*_1_ = 0.1 V.

Differential common-source structures [32,33] and stacked structures [34,35] are two circuit topologies commonly used in PA design. The differential common-source structure can suppress even harmonics. In contrast, the stacked structure can achieve a large output voltage swing (i.e., the transistors are connected in series to achieve a superposition of bias voltages and increase the output voltage swing of the PA) while ensuring that the transistors do not break down, thus increasing the output power of the PA. We designed and implemented a differential triple-stacked millimeter-wave broadband PA with on-chip temperature compensation based on the differential common-source structure and stacked structure.

The PA uses a two-way differential stacking structure to increase the output power and improve the stability of the PA by introducing a negative feedback network. To increase the bandwidth and reduce losses, on-chip transformers were used for the matching networks at the input/output of the PAs designed in this study. A thick metal layer was used in the design process for the on-chip transformer and interconnecting lines. As shown in Figure 3, the gate width/gate length of transistors M_CG2_, M_CG1_, and M_CS_ were 4 × 60 μm/60 nm, 4 × 60 μm/60 nm, and 3 × 60 μm/60 nm, respectively. Meanwhile, to improve power gain, isolation, and gain flatness, the neutralizing capacitor (C_n_), shunt capacitor (C_in_), and shunt resistor (R_g_), respectively, were introduced in this study.

**Figure 4:**
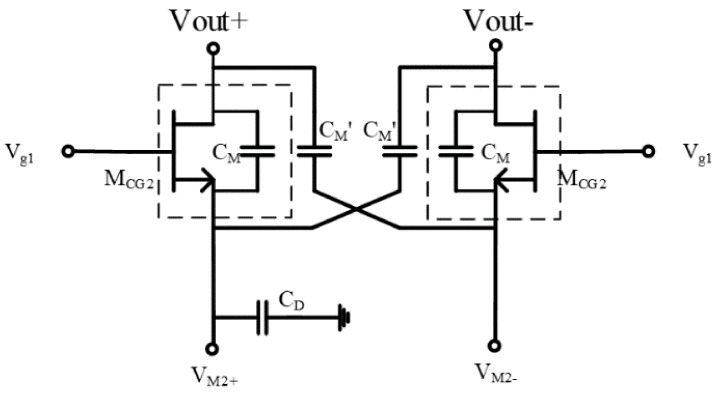
Schematic diagram of the negative feedback network.

Although introducing a neutralization capacitor (C_n_) can improve the stability of a PA to some extent, the PA will still be unstable when the parasitic capacitance (C_M_) between the source and drain of transistor M_CG2_ is relatively large. This is also one of the main reasons for the instability of the stacked structure amplifier. Therefore, as shown in Figure 4, this study introduced another capacitor (C_M_’) in the negative feedback network of the top layer in order to improve stability. The capacitor (C_D_) is the raw capacitance of the drain of transistor, M_CG1_. The regulation of C_M_’ is used to achieve a balance between the three capacitors C_M_’, C_M_, and C_D_ in order to achieve the stability of the PA.

**Figure 5:**
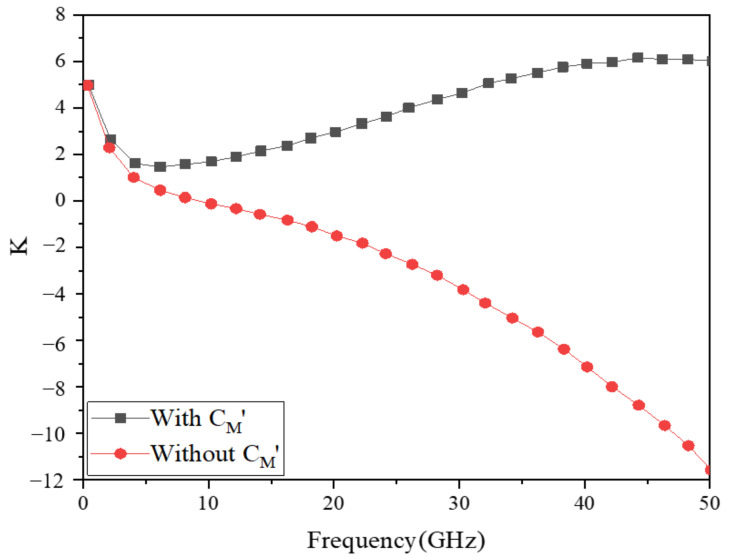
Stability factor of PA.

The stability of the PA is shown in Figure 5. According to Figure 5, this PA’s stability factor (K) in the range of 0–5 GHz was greater than 1 without introducing C_M_’, and the PA was in a stable state. The StabFact in the other frequency ranges was less than 1, and the PA was in an unstable condition. When C_M_’ was introduced into the PA, the stability factor of the PA in the whole frequency range was always greater than 1. As a result, the PA remained stable over the entire operating frequency range. This indicates that the introduction of capacitance (C_M_’) in the negative feedback network of the top layer of this stacked PA can effectively improve the stability of the PA.

**Figure 6:**
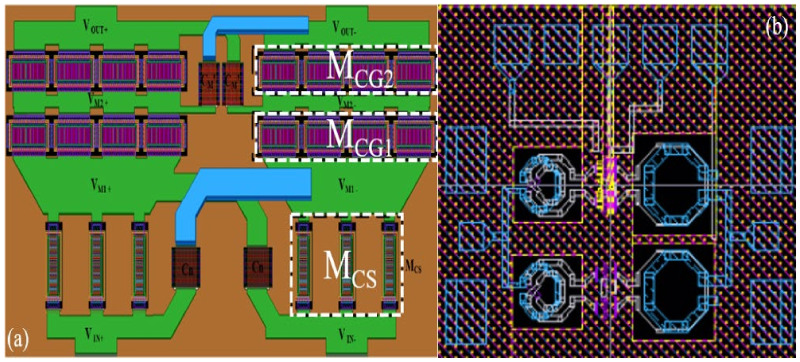
PA’s overall layout and core layout: (**a**) core layout; (**b**) overall layout.

Figure 6 shows the core layout and overall layout of a differential stacked millimeter-wave wideband PA. The layout of the PA has a significant impact on the performance of the PA. Therefore, to simplify the RF signal path to reduce power consumption, we kept the layout of transistors M_CG1_ and M_CG2_ in the stacked structure, shown Figure 3, perpendicular to the direction of transistor M_CS_ when laying out the PA. The overall area of the PA chip was 0.378 mm^2^.

## 5. Results and Discussion

### 5.1. Small-Signal Gain at Different Temperatures

**Figure 7:**
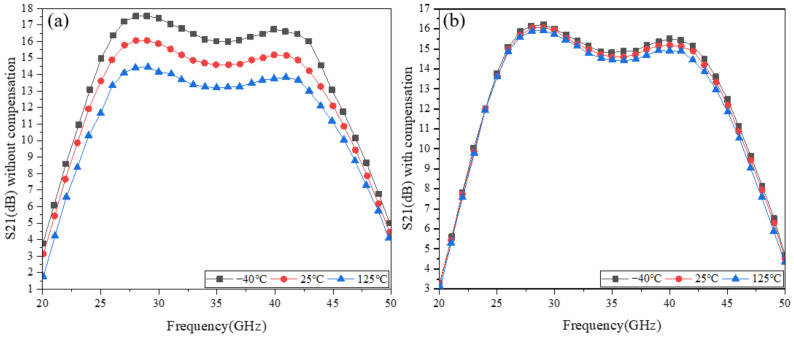
Small-signal gain versus temperature variation: (**a**) without temperature compensation; (**b**) with temperature compensation.

Figure 7 gives the small-signal gain versus temperature variation for the differential stacked millimeter-wave broadband power amplifier. Figure 7a,b show the results without and with temperature compensation circuits, respectively.

As seen in Figure 7, without the compensation, the small-signal gain varied by 2.4 dB in the frequency range of 25–40 GHz, while with the compensation, the small-signal gain varied by 0.1 dB in the frequency range of 25–40 GHz. The specific reasons are discussed below.

The small-signal gain is the transconductance [29], and the expression for the transconductance is

(13) $$g_{m}=\sqrt{2\mu_{n}C_{ox}\frac{W}{L}I_{ds}},$$

and the main factors affecting the transconductance are *μ_n_* and *I_ds_*, where the expression for the drain current in the saturation region is [36]

(14) $$I_{ds}=\mu_{n}C_{ox}(\frac{W}{L}){\left(V_{gs}-V_{th}\right)}^{2},$$

where *V_gs_* is the gate voltage, and *V_th_* is the threshold voltage.

When *V_gs_* increases, Ids also increases with the rise of *V_gs_*. It is also known from Equation (13) that the small-signal gain increases as *I_ds_* increases. When the temperature rises, *μ_n_* decreases with the increase in temperature, resulting in a reduction in small-signal gain. With the compensation, *I_ds_* increases as the temperature rises to effectively compensate for the degradation of the small-signal gain due to *μ_n_*. In other words, the gate voltage *V_g_*_1_, gate voltage *V_g_*_2_, and gate voltage *V_g_*_3_ increase with the temperature, which increases the *I_ds_*, and thus, compensates for the small-signal gain. This indicates that the on-chip temperature compensation circuit has a good compensation effect on the small-signal gain.

### 5.2. Output Power and PAE at Different Temperatures

**Figure 8:**
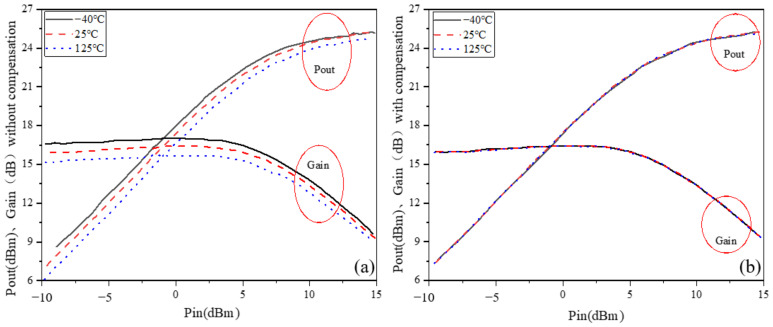
Output power versus temperature variation: (**a**) without temperature compensation; (**b**) with temperature compensation.

The output power (P_out_) and large-signal gain at the center frequency of the PA versus temperature are shown in Figure 8. As shown in Figure 8a, the P_out_ and large-signal gain with an on-chip temperature compensation circuit varied very little. In contrast, the PA’s output power and large-signal gain without a temperature compensation circuit were more significantly affected by temperature variations.

**Figure 9:**
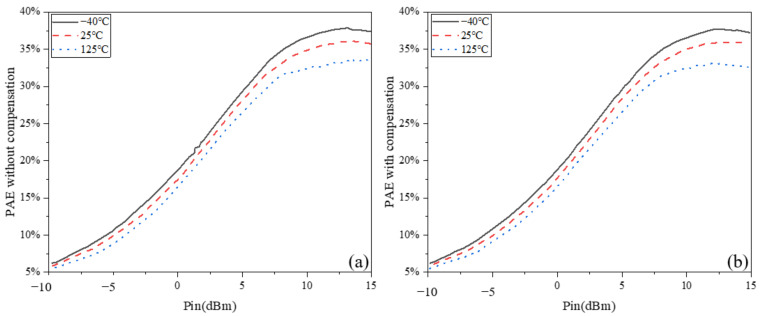
PAE versus temperature variation: (**a**) without temperature compensation; (**b**) with temperature compensation.

The power-added efficiency (PAE) at the center frequency of the PA versus temperature is shown in Figure 9. It is worth noting that the effect of temperature on the PA’s power-added efficiency was relatively large, regardless of whether it was with compensation. This indicates that the compensation circuit has a better compensation effect on the P_out_ and large-signal gain, while the compensation effect on the PAE is insignificant. The specific reasons are discussed below.

Literature studies have shown [37] that the on-state resistance between source and drain increases with temperature, which leads to a drop in the P_out_. The expression for on-resistance is [37]

(15) $$R_{on}=R_{0}+kT^{\alpha }/{\left|V_{gs}-V_{th}\right|}^{\beta },$$

where the constant *k* is a process parameter, *R*_0_ is a constant independent of voltage and temperature, *α* ≈ 1.5, *β* ≈ 0.2, *V_gs_* is the gate voltage, and *V_th_* is the threshold voltage.

According to Equation (15), on-resistance increases with increasing temperature. Therefore, when the PA does not have a temperature compensation circuit, the P_out_ decreases with increasing temperature. On the other hand, when the PA is with compensation, the gate *V_gs_* in Equation (15) increases with the temperature, causing the on-resistance to decrease with the gate voltage increase, thus achieving the compensation for the P_out_. As a result, the compensation for the large-signal gain is also achieved. As for the PAE, the compensation circuit compensates for the P_out_ while increasing the DC power consumption of the PA [24]. Therefore, according to the definition of PAE, the PAE decreases with the increase in DC power consumption. Thus, with or without compensation, the effect of temperature change on PAE of PA is relatively significant [24].

Table 1 compares the amplifier’s performance with on-chip temperature compensation circuits. Compared with the compensation circuit structures and methods in the literature [23,24,38,39,40], the temperature compensation circuit proposed in this paper has a simple structure, a smaller overall chip area, and a better compensation effect for small-signal gain and output power. Still, the compensation effect for PAE was poor. In addition, these post-layout simulation results can be regarded as a supplement to the measured results in Refs. [23,38,39,40]. This idea is consistent with Ref. [24], and these results are effective in the relative amount of change in small-signal gain, output power, and PAE before and after compensation.

Furthermore, in terms of the magnitude of small-signal gain variation with temperature, the compensation circuit can effectively compensate for the small-signal gain. This shows that the proposed compensation circuit for differential stacked millimeter-wave wideband PAs effectively compensates for the degradation of the small-signal gain of PAs due to temperature variations over a wide range.

## 6. Conclusions

This paper proposes a compensation circuit consisting of two diodes and four resistors for a PA with a stacked structure. To verify the effectiveness of the compensation circuit, we designed and implemented a differential triple-layer stacked structure millimeter-wave wideband PA.

With the temperature-compensation circuit, the small-signal gain variation improved from 2.4 to 0.1 dB in the temperature range between −40 °C and 125 °C. It was demonstrated that the proposed on-chip temperature compensation circuit effectively corrects the small-signal gain, large-signal gain, and P_out_ variations of a millimeter-wave PA with a multi-layer stacked structure over a wide temperature range. In addition, the proposed on-chip temperature compensation circuit also applies to multi-stage cascaded microwave/mm-wave power amplifiers.

## Figures and Tables

**Table 1:** Performance comparison with previous works.

Reference	Ref. [23]	Ref. [38]	Ref. [39]	Ref. [40]	Ref. [24]	This Work ^②^
Circuit	PA	LNA	LNA	PA	PA	PA
Technology	90 nm	130 nm SOI	130 nm	40 nm	GaN HEMT	55 nm
Topology	2-stage	1-stage	1-stage	3-stage	1-stage	1-stage
Frequency (GHz)	26.5	2.4	15.2	79	4.5–5.5	25–40
Small-signal gain variation (dB)	1.2 (−45~125 °C)	0.9 (25~200 °C)	3.1 (−20~120 °C)	0.6 (10~100 °C)	0.4 (−40~225 °C)	0.1 (−40~125 °C)
Pout variation (dBm) (center frequency)	N/A	N/A	N/A	N/A	24.0 (−40~225 °C)	0.6 (−40~125 °C)
PAE variation (center frequency)	N/A	N/A	N/A	N/A	12% (−40~225 °C)	7.3% (−40~125 °C)
Area (mm^2^)	0.5 ^①^	0.6 ^①^	0.6 ^①^	0.11 ^①^	6.6	0.378

Note: ^①^ DC pads are not included. ^②^ Post-layout simulation results.
